# Diagnosis-Specific Links Between Physical Activity and Sleep Duration in Youth with Disabilities: A Systematic Review with Quantitative Synthesis

**DOI:** 10.3390/ijerph23010121

**Published:** 2026-01-19

**Authors:** Janette M. Watkins, Martin E. Block, Janelle M. Goss, Emily M. Munn, Devan X. Antczak

**Affiliations:** 1 Department of Kinesiology, College of Health and Human Development, The Pennsylvania State University, University Park, PA 16802, USA; jfg6171@psu.edu; 2 Department of Education, University of Virginia, Charlottesville, VA 22904, USA; meb7u@virginia.edu; 3 Department of Kinesiology, School of Public Health Bloomington, Indiana University, Bloomington, IN 47405, USA; emmunn@iu.edu; 4 Department of Kinesiology, School of Education, University of Wollongong, Wollongong, NSW 2522, Australia; devan@uow.edu.au

**Keywords:** chronic health conditions, diagnosis-specific interventions, disability-inclusive health promotion, sleep, physical activity

## Abstract

**Highlights:**

**Public health relevance—How does this work relate to a public health issue?**

- Children and adolescents with disabilities experience disproportionately low physical activity and inadequate sleep, two behaviors central to lifelong health and functioning.
- This review addresses a critical gap in population health evidence by examining how physical activity and sleep are linked across diverse disability groups.

**Public health significance—Why is this work of significance to public health?**

- Diagnosis type was a key determinant of sleep duration, revealing substantial heterogeneity in sleep health among youth with disabilities.
- Meeting physical activity guidelines may support longer sleep duration among youth with autism spectrum disorder, identifying physical activity as a potentially modifiable behavior for improving sleep health.

**Public health implications—What are the key implications or messages for practitioners, policy makers, and/or researchers in public health?**

- Public health strategies should prioritize accessible, diagnosis-specific physical activity opportunities within schools, communities, and clinical care settings to support sleep health.
- Researchers and policy makers should move beyond one-size-fits-all recommendations and consider diagnosis-specific approaches when developing inclusive physical activity and sleep interventions.

**Abstract:**

Children and adolescents with disabilities experience disproportionate challenges in achieving recommended levels of physical activity (PA) and adequate sleep, two core determinants of health and functional well-being. This systematic review examined associations between meeting PA guidelines and sleep duration among youth with disabilities. Following PRISMA guidelines, MEDLINE, PsycARTICLES, and SPORTDiscus were searched through Spring 2024 for studies assessing PA and sleep in children and adolescents (<18 years) with disabilities using subjective or objective measures. Data were extracted from 28 studies (*N* = 138,016) and synthesized using qualitative methods and regression-based quantitative analyses to examine the effects of diagnosis category and PA guideline adherence on sleep duration. The diagnosis type was associated with sleep duration, with youth with autism spectrum disorder (ASD) exhibiting shorter sleep than those with physical disabilities. Meeting PA guidelines (≥60 min/day) was associated with longer sleep duration among youth with ASD, but not consistently across other diagnostic groups. Qualitative findings further indicated diagnosis-specific variability, with PA positively associated with sleep outcomes in ASD, attention deficit/hyperactivity disorder, and epilepsy, and mixed associations observed for cerebral palsy and intellectual disability. These findings suggest that PA may support sleep health in specific disability groups. Given persistently low PA participation among youth with disabilities, integrating accessible, diagnosis-specific PA opportunities within school, community, and clinical settings may represent a feasible strategy to improve sleep and overall health.

## 1. Introduction

The daily recommendation for children and adolescents to engage in 60 min or more of moderate-to-vigorous physical activity (MVPA) has known health benefits [1]. Alongside physical health improvements, regular physical activity (PA) has been linked to better sleep in adults without disabilities [2]. Other researchers conducted a meta-analysis focusing on PA and sleep in children without disabilities [3], finding that PA correlated with longer sleep duration, aligning with adult studies. However, there is limited research exploring this link in children and adults with disabilities [4], even though these populations often face challenges in meeting PA guidelines compared to their counterparts without disabilities [5,6].

Studies have consistently shown that children with disabilities, such as intellectual disability, physical disabilities, and neuropsychological disorders, tend to engage in less MVPA than their peers without these conditions [5,7]. They also frequently fall short of meeting recommended PA levels [7,8]. Specific conditions like attention deficit hyperactivity disorder (ADHD), autism spectrum disorder (ASD), cerebral palsy (CP), cystic fibrosis (CF), and intellectual disability (ID) have all been associated with inadequate PA levels [9,10,11,12].

Consequently, sleep issues are prevalent among children with disabilities, with reduced sleep duration being a common concern [13,14]. Sleep problems are consistently reported across various disorders like ADHD [15], ASD [16], CP [17], CF [18], ID [19], Duchenne muscular disorder (DMD) [20], Down syndrome (DS) [21], epilepsy [22], and Tourette’s syndrome [23]. These sleep difficulties often exacerbate behavioral issues and limit daily activities in children and adolescents with and without disabilities [24,25]. They are also linked to a decreased quality of life for affected children and adolescents [26,27,28]. Understanding the relationship between PA and sleep outcomes is crucial, as PA may serve as a potential strategy to improve sleep quality and mitigate associated behavioral problems in these populations.

Despite the significant impact of PA on sleep in children without disabilities [3], this relationship remains understudied in children with disabilities. While there are reports on PA levels and sleep outcomes in these populations, there’s a gap in understanding how physical activity and sleep outcomes are associated across disability groups. A few studies have suggested a positive connection between high PA and better sleep among children with ADHD and ASD [29,30], but consistent findings across different disabilities are lacking. Therefore, the objective of this systematic review was to comprehensively evaluate the relationship between physical activity and sleep outcomes among children and adolescents with disabilities. Specifically, we aimed to (1) determine whether meeting physical activity guidelines is associated with sleep duration and quality across disability types at the study level, and (2) identify diagnosis-specific patterns that may inform tailored physical activity interventions to improve sleep health in this population.

## 2. Materials and Methods

### 2.1. Eligibility Criteria

To be included in this review, studies had to meet four criteria: (1) being published in a peer-reviewed English-language journal, (2) assessing PA and sleep using objective or subjective measures, (3) including children and adolescents with disabilities in the study sample, and (4) involving participants under 18 years old. Cross-sectional and experimental designs in the review were included. While many articles included PA and sleep as variables, not all examined the direct relationship between them. For studies to be included in the quantitative synthesis, they needed to provide unadjusted values for PA and sleep, such as means with standard deviations or the percentage of participants meeting PA/sleep guidelines. To be included in the qualitative analysis, studies needed to provide knowledge on the direct relationship between PA and sleep.

### 2.2. Outcomes of Interest

The primary outcomes of interest for this systematic review were measures of sleep and PA. Sleep duration refers to the total amount of sleep obtained in a 24 h period. Measures of PA included total PA, total MVPA, and the percentage of participants meeting PA recommendations. The review aimed to explore how PA and sleep outcomes are related within and across different disabilities, examining whether PA can potentially improve sleep outcomes. The review also sought to identify any differences in the relationship between PA and sleep outcomes based on the type of disability, age, and severity of the disability.

### 2.3. Search Strategy

We conducted a systematic review following the Preferred Reporting Items for Systematic Reviews and Meta-Analyses (PRISMA) guidelines [31]. The initial search was conducted during the fall semester of 2022, followed by a subsequent search in the spring semester of 2024, encompassing all articles published up to that date. We searched the following databases: MEDLINE, APA PsycARTICLES, and SPORTDiscus. Our search strategy combined terms related to age groups (child*, youth, teen*), topics of interest (sleep*, physical activit*), and the specific population (disabilit*). This approach aimed to comprehensively identify relevant literature on the relationship between sleep, physical activity, and disabilities among children and youth. This systematic review was registered with the International Prospective Register of Systematic Reviews (PROSPERO; registration number: CRD420251274807).

### 2.4. Selection Process and Data Extraction

Two independent reviewers screened each record with a title and abstract and conducted the full-text screening. Consistent with the PRISMA statement for reporting systematic reviews, two authors (JW and JG) extracted the following data from each study that met inclusion: sample size, participant characteristics (e.g., age, sex), diagnosis type, description of the objective or subjective measurement of independent and dependent variables, and sleep and physical activity outcomes. These details are provided in Table 1.

### 2.5. Quality Assessment Strategy

The quality of the included studies was assessed using the AXIS risk of bias tool [58]. The AXIS tool examines several aspects, including the clarity of study objectives, appropriateness of study design, selection process of participants, measurement of variables, and potential sources of bias. Each study was evaluated based on these criteria to determine the risk of bias and overall methodological rigor. Studies were rated on aspects such as the clarity of reporting, the robustness of statistical analysis, and the extent to which confounding factors were addressed. This rigorous assessment helped ensure that the findings of the review are based on high-quality evidence and reliable data. Quality assessment was independently conducted by two authors (JW and JG), achieving high inter-rater reliability (94%, with discussions achieving full agreement (100%).

### 2.6. Statistical Analysis

Due to substantial heterogeneity across studies in disability classification, study design, and physical activity and sleep measurement, a traditional effect-size–based meta-analysis was not feasible. Many included studies did not report standardized effect estimates or sufficient data to derive comparable effect sizes. This limitation is common in public health and disability research, where outcomes are frequently reported as study-level means or proportions meeting guidelines [5,59,60]. Accordingly, we conducted an exploratory regression-based quantitative synthesis using aggregated study-level data to examine study-level associations between diagnosis category, physical activity guideline adherence, and sleep duration. Similar approaches have been used in heterogeneous public health reviews to identify population-level patterns when effect-size harmonization is not possible [59,61]. This strategy enables inclusion of a broader evidence base while preserving diagnostic specificity and supporting translation to real-world, population-focused intervention contexts. Statistical analyses were performed using RStudio (2024.04.2+764). Linear regression models were employed to investigate whether participant diagnosis and physical activity category (meeting recommendations vs. not meeting recommendations) could predict sleep duration. Conditions with very limited representation were grouped into the Physical Disabilities category to allow for inclusion in the exploratory quantitative synthesis; this grouping was not intended to imply clinical or mechanistic homogeneity. Model selection was determined by assessing significant differences in model fit through an analysis of variance. Significant effects were further analyzed using a multiple comparisons test with a Bonferroni adjustment. The threshold for statistical significance was set at *p* < 0.05. Although the total number of participants across studies was large, all quantitative analyses were conducted using study-level observations; therefore, the findings reflect patterns across studies rather than individual-level effects.

## 3. Results

### 3.1. This Study Selection

**Figure 1:**
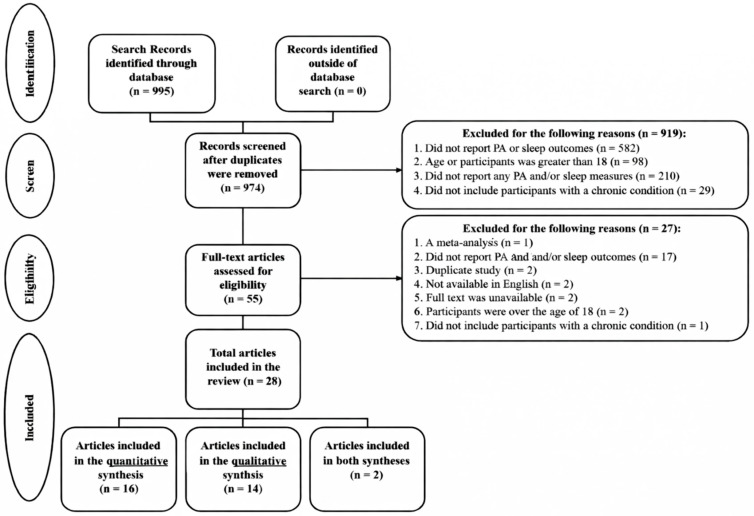
PRISMA flow diagram.

As shown in Figure 1, a total of 995 records were identified and 974 records were retained after duplicates were removed. Most of these studies were excluded for various reasons (see Figure 1), resulting in 14 articles included in the qualitative synthesis and 16 were in the quantitative synthesis. Inter-rater agreement from all data extracted from included studies was high (*M* = 96.5%, rang = 83.7–100%). Any disagreements of inclusion and exclusion were settled by discussion.

### 3.2. Study Description

Table 1 provides an overview of the characteristics of all studies included in the review. Out of the 28 studies, 24 provided information on gender, totaling 138,016 participants: 40,404 females, 40,365 males, and 57,247 with gender not being specified. The review encompassed 52,841 participants with disabilities across 11 different diagnoses. These conditions included ASD in nine studies [14,29,32,35,36,48,53,55,62] with 30,361 participants, attention deficit/hyperactivity disorder (ADHD) in six studies [14,40,41,44,45,49] with 7447 participants, cerebral palsy (CP) in five studies [38,42,46,52,56] with 293 participants, cystic fibrosis (CF) in one study [47] with 28 participants, depression in three studies with 6108 participants, Duchenne muscular dystrophy (DMD) in one study[51] with 54 participants, Down syndrome (DS) in one study [14] with 50 participants, epilepsy in three studies [14,34,50] with 7367 participants, intellectual disability (ID) in four studies [14,43,54,57] with 1285 participants, Tourette’s syndrome in one study [49] with 110 participants, and visual impairments [39] in one study with 561 participants.

Specifically focusing on ID, four studies [14,43,54,57] contributed data on 1285 participants, comprising 330 females, 842 males, and 113 with unreported gender. Tourette’s syndrome was addressed in a study [49] involving 110 participants (24 females and 86 males), while visual impairments were explored in a study [39] with 561 participants (314 females and 247 males).

Sleep outcomes were assessed using subjective methods such as questionnaires (*n* = 8), sleep logs (*n* = 4), and recall (*n* = 2). Objective measurements were obtained through electroencephalogram (*n* = 1), actigraphy (*n* = 2), and other accelerometers (*n* = 13). The sleep outcomes investigated included disturbed sleep (*n* = 6), time to fall asleep (*n* = 4), sleep duration (*n* = 13), and sleep quality (*n* = 5). Sleep duration was chosen as the primary outcome due to its prevalence as the most consistently reported sleep measure across the included studies. Supplementary Materials File S1 shows all studies and their unique methods and characteristics pertaining to sleep and PA.

### 3.3. Quality Assessment

**Figure 2:**
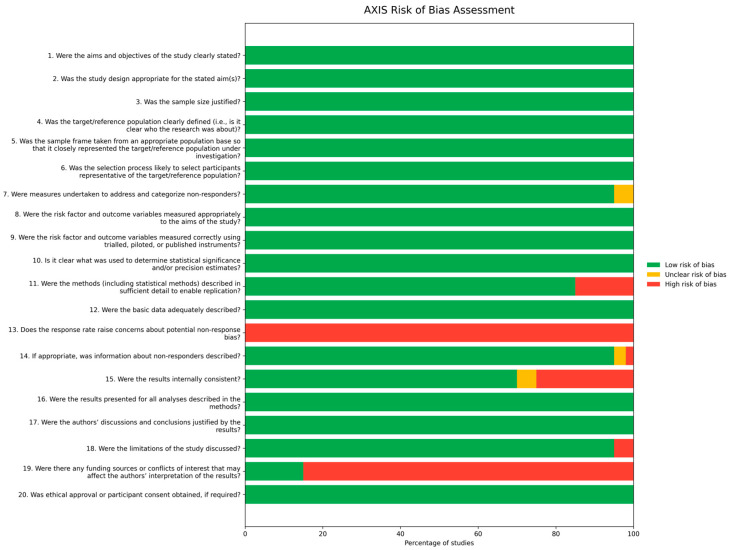
Risk of bias summary.

Out of the total number of studies, 23 were categorized by the AXIS tool as having a low risk of bias, and 5 studies were identified as having a medium risk of bias [14,29,43,45,62]. The primary sources of bias were typically related to inadequate reporting on non-responders, insufficient justification for sample size, and a lack of detailed description regarding basic data needed for replication purposes. See Figure 2 for more detail.

### 3.4. Methodological Characteristics and Quality of Included Studies

Across the included studies, substantial variability was observed in the sample size, study design, and measurement approaches. Sample sizes ranged from fewer than 30 participants in condition-specific intervention studies to over 20,000 participants in population-based surveys. Smaller studies were more common among physically disabling conditions (e.g., cystic fibrosis, Duchenne muscular dystrophy), whereas autism spectrum disorder and ADHD were more frequently represented in large epidemiological datasets. Physical activity was assessed using a wide range of methods, including accelerometry, parent-report questionnaires, self-report surveys, and proxy measures such as guideline adherence, limiting cross-study comparability. Sleep outcomes were similarly heterogeneous, with studies reporting total sleep duration, sleep efficiency, sleep onset latency, or subjective sleep quality. Most studies employed cross-sectional designs, with relatively few longitudinal or intervention-based studies, limiting causal inference. Collectively, these methodological patterns highlight the need for larger, diagnosis-specific studies using standardized and objective measures of both physical activity and sleep, as well as longitudinal designs capable of clarifying temporal relationships.

### 3.5. Qualitative Analysis

Table 2 presents key findings from studies investigating the association between PA and sleep measures across various conditions. Studies were only included in Table 2 if they provided knowledge on the relationship between PA and sleep (*N* = 14). Additional detail concerning PA and sleep recommendations can be found below.

Studies indicated that individuals with ASD often fail to meet recommended PA and sleep levels compared to peers without disabilities [14,48,62]. Higher levels of moderate-to-vigorous activity correlated with improved sleep [62], and PA was found to enhance sleep outcomes [29,32]. Regarding neurological conditions like ADHD, individuals with ADHD were observed to fall short of recommended sleep and PA levels compared to non-ADHD individuals [14,40], noting that PA may improve sleep outcomes and overall quality of life in this population. For epilepsy, children were less likely to meet PA recommendations [14,34,50], but those meeting recommendations showed improved sleep outcomes [34]. Individuals with Depression were also less likely to meet PA and sleep recommendations, experiencing poorer sleep quality and increased depressive symptoms [14,33,49]. Physical disabilities such as CP [38,42,52], CF [47], DMD [51], and visual impairments [39] were studied for their impact on PA and sleep. Two studies found that individuals with CP were less active, with sedentary behavior influencing sleep quality [42,52]. However, another study examining CP found an increase of PA levels decreased total sleep duration [38]. In CF, sedentary time was linked to poor sleep [47]. DMD research suggested a link between rest and activity levels and subjective sleep impairment in ambulatory cases [51], although further research is needed. Among individuals with ID, there was a possible link between sleep duration/timing and PA, with earlier wake times supporting more PA [54].

### 3.6. Quantitative Analysis

Sixteen unique studies contributed to the quantitative synthesis. Three studies contributed two independent study-level data points because they reported sleep and physical activity outcomes separately for distinct subgroups (e.g., by diagnosis or physical activity category), resulting in a total of 22 study-level observations included in the regression models.

An exploratory general linear regression model was used to examine study-level associations between physical activity (PA) category (less than 60 min daily vs. 60 min or more daily), diagnosis category, and average sleep duration. Diagnosis categories included autism spectrum disorder (ASD), intellectual disability (ID; encompassing Down syndrome and other ID diagnoses), physical disabilities (including cystic fibrosis, cerebral palsy, Duchenne muscular dystrophy, and visual impairments), and neurological conditions (epilepsy, ADHD, and depression). Studies classified under ID reported aggregated outcomes across intellectual disabilities without etiological differentiation (e.g., Down syndrome vs. other causes); findings should therefore be interpreted as population-level descriptors rather than diagnosis-specific estimates.

**Figure 3:**
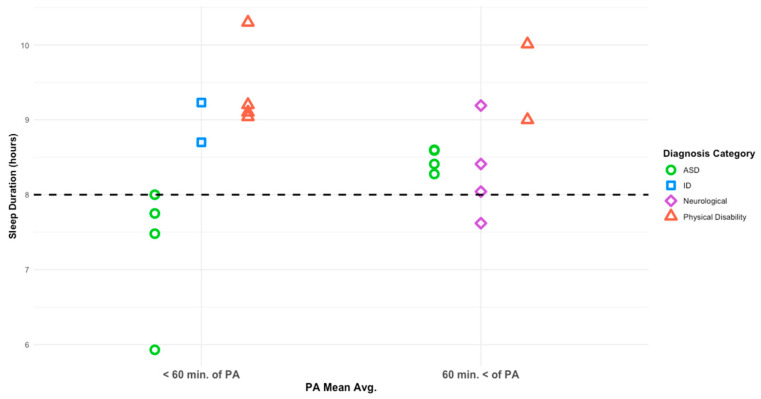
Average sleep duration based on PA mean per diagnosis category. The dashed line denotes the recommended 8 h of nightly sleep.

**Figure 4:**
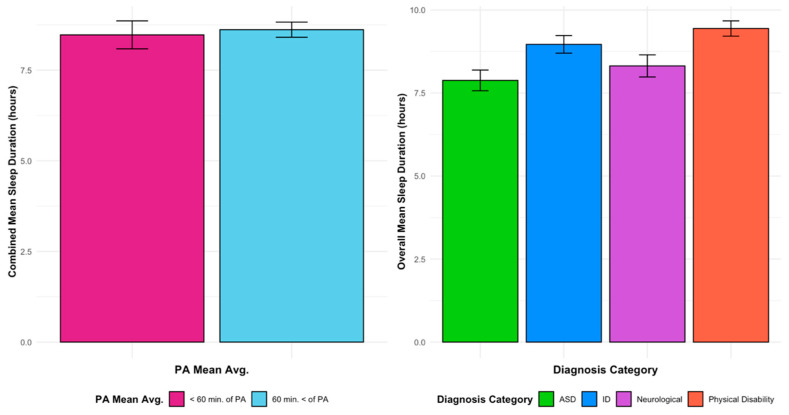
Sleep duration vs. PA means for all diagnoses vs. diagnoses and sleep means.

Diagnosis category was associated with sleep duration at the study level (F(3,18) = 6.742, *p* = 0.006). Physical activity category was not a statistically significant predictor of sleep duration (F(1,18) = 3.569, *p* = 0.083), with relatively small differences observed between studies in which participants met PA guidelines (M = 8.9 h, SD = 0.264) and those in which participants did not (M = 8.5 h, SD = 0.225). The interaction between PA category and diagnosis category was not significant (F(1,18) = 0.643, *p* = 0.438). Covariates including mean age (F(1,18) = 2.124, *p* = 0.176) and study sample size (F(1,18) = 0.274, *p* = 0.612) did not significantly contribute to the model. Study-level patterns of average sleep duration by diagnosis category and physical activity adherence are presented in Figure 3. Figure 4 depicts study-level associations between mean physical activity and sleep duration across diagnosis categories.

Post-hoc multiple comparisons indicated that studies categorized under ASD reported shorter average sleep duration (M = 7.8 h, SD = 0.229) than those categorized under physical disabilities (M = 9.7 h, SD = 0.374), with a mean difference of 1.89 h (SD = 0.438, *p* = 0.001, 95% CI: 0.60, 2.95). Comparisons between neurological conditions (M = 8.3 h, SD = 0.324) and physical disabilities approached statistical significance (mean difference = 1.34 h, SD = 0.458, *p* = 0.054).

Within the ASD category, pairwise comparisons of least-squares means demonstrated that studies in which participants met PA guidelines reported longer average sleep duration than those in which participants did not (M = 9.0 h, SD = 0.459 vs. M = 7.8 h, SD = 0.229; *p* = 0.024). These findings reflect study-level patterns suggesting a nuanced relationship between physical activity, diagnosis category, and sleep duration, and should be interpreted as exploratory rather than causal or individual-level effects.

## 4. Discussion

### 4.1. Summary

The aim of this systematic review was to evaluate the relationship between PA and sleep outcomes in children and adolescents with disabilities, building upon existing research in children without disabilities [3]. Through this analysis, there were two primary quantitative findings: (1) diagnosis category was associated with differences in average sleep duration at the study level notably highlighting that children and adolescence with ASD have the shortest sleep duration, and (2) within the ASD population, adhering to PA guidelines (i.e., more than 60 min of PA daily) may increase sleep duration. Key qualitative findings supported these findings, with varied impacts of physical activity on sleep quality and duration across different health conditions, emphasizing the need for tailored interventions to optimize outcomes.

### 4.2. Diagnosis-Specific Associations Between PA and Sleep

One key finding from the quantitative synthesis was that diagnosis category was associated with differences in average sleep duration at the study level, highlighting variability in sleep patterns across health conditions. Specifically, studies involving youth with ASD reported shorter average sleep duration compared to studies categorized under physical disabilities [14,62]. However, these findings should be interpreted cautiously given the study-level design and limited number of observations. Poor sleep outcomes are reported in other research, with children and adolescents with ASD showing a 13% prevalence of sleep disorders compared to typically developing peers (3.7%) [63]. Indeed, sleep disorders are the most common complaints reported in individuals with an ASD diagnosis [64]. Importantly, sleep disturbances in children and adolescents with physical disabilities are less studied than those with ASD. Most research focuses on caregivers of children with physical disabilities, rather than the children and adolescents themselves [65,66,67]. This gap in research highlights the need for more studies directly assessing sleep issues in children and adolescents with physical disabilities. The Physical Disabilities category encompassed heterogeneous conditions, many represented by a single study. As such, quantitative comparisons involving this category should be interpreted cautiously and viewed as descriptive rather than generalizable across physical disability diagnoses. Understanding the unique sleep challenges for each diagnostic group is important for developing effective, tailored interventions. The second key finding was that adhering to PA guidelines correlated to increased sleep duration in children and adolescents with ASD. Within the ASD category, higher PA engagement was associated with longer nightly sleep durations [32,55], highlighting the potential benefits of structured PA programs in improving sleep outcomes for this population. This finding is important, given that only 42% of people with ASD are predicted to meet PA recommendations [68], with a key barrier to PA participation being lack of tailored programs [69]. These findings suggest that tailored PA interventions that consider diagnosis-specific needs could effectively support sleep health in clinical practice. This is consistent with other literature that suggests in children and adolescent populations, tailored or personalized PA programs are more successful in promoting health outcomes including sleep quality [70,71]. Future research could further explore individualized strategies integrating PA into comprehensive care plans to optimize sleep and overall well-being for children and adolescents managing disabilities.

Qualitatively, similar findings emerged, indicating variability in the relationship between PA and sleep outcomes across different diagnosis categories. Studies across different populations highlight that, while regular PA generally enhances sleep quality and duration, these benefits can vary depending on specific disability and associated factors. For example, for individuals with ASD, factors such as the severity of ASD, gender differences, and age moderated the effects of PA on sleep [35]. This is supported by other empirical work in populations without disabilities, where age [2], gender [72] and other individual differences [71] influence the relationship between sleep and PA. However, future research is needed to better assess the potential effects of individual variations in populations with disabilities.

Each diagnosis was explored in more detail for the relationship between sleep and PA. For conditions like CF [9,18], DMD [51], epilepsy [34], and ADHD [30,40], PA appears to positively influence sleep quality. This is consistent with other empirical work which found that PA (particularly sport-based activities) is associated with better sleep levels children and adolescents without disabilities [73]. For individuals with ID, research suggests that sleep duration may influence PA levels, with early risers generally exhibiting higher activity levels [54]. This finding is supported by work in typically developing children, which suggests that exercise time is related to sleep duration and efficiency, with morning exercise promoting better sleep outcomes [74]. Moreover, despite typically engaging in less PA than their peers, individuals with CP do not consistently exhibit differences in sleep duration compared to counterparts without disabilities [26,56]. Interestingly, increased sedentary time rather than reduced PA seems to be associated with poorer sleep outcomes in this group [52]. This relationship is also seen in other systematic reviews in adults without disabilities [37] and children [75], with high daily sedentary time associated with poor sleep outcomes. This may be due to prolonged sedentary behavior disrupting circadian rhythms and contributing to sleep disturbances by altering melatonin production and increasing arousal levels close to bedtime [76]. Moreover, sedentary behaviors such as excessive screen time have been linked to physiological changes that may negatively impact sleep quality and duration [77]. To mitigate these effects, promoting smaller bouts of PA throughout the day could be beneficial. Studies suggest that even brief periods of physical activity, such as brain breaks or short walks, can counteract the negative impacts of sedentary behavior on sleep [76]. These interventions not only break up prolonged sitting but also contribute to overall physical and mental well-being.

### 4.3. Public Health Implications

From a public health perspective, these findings underscore the importance of addressing physical activity and sleep as interconnected, modifiable behaviors among children and adolescents with disabilities. Low rates of physical activity guideline adherence in this population highlight the need for scalable, disability-inclusive strategies that extend beyond clinical care into schools, community programs, and adapted physical education settings. The diagnosis-specific associations observed—particularly among youth with ASD—suggest that one-size-fits-all approaches may be insufficient and that tailored physical activity opportunities may yield greater downstream benefits for sleep health. Integrating accessible physical activity programming within daily routines has the potential to support sleep duration, overall health, and quality of life at the population level, while reducing persistent health disparities experienced by youth with disabilities.

These findings are also relevant within the context of international disability policy. The United Nations Convention on the Rights of Persons with Disabilities (UN CRPD) affirms the right of persons with disabilities to the highest attainable standard of health (Article 25) and to participation in recreational, leisure, and sporting activities (Article 30.5). Notably, Article 25.b emphasizes that States Parties are obligated to provide health services needed specifically because of disability. Within this framework, equitable access to inclusive and diagnosis-appropriate physical activity opportunities may be viewed not only as a public health priority, but also as a rights-based obligation that supports sleep health and overall well-being among children and adolescents with disabilities.

### 4.4. Limitations

One limitation of this review was the inadequate availability of research for certain diagnoses. For example, conditions such as cystic fibrosis and visual impairment were each represented by a single study, with the small sample size of the cystic fibrosis study (*n* = 28) posing challenges for drawing definitive conclusions about this subgroup. Moreover, the overall body of evidence was limited in size (*n* = 28 studies). To strengthen the evidence base supporting the potential role of physical activity in sleep health among youth with disabilities, additional empirical studies are needed. The relationship between physical activity and sleep remains mixed across diagnoses, and future research should aim to address this gap through larger, diagnosis-specific investigations using harmonized measurement approaches. Increasing the number of studies directly examining physical activity–sleep relationships in children and adolescents with disabilities will provide a more robust foundation for understanding potential benefits and mechanisms. Additionally, the heterogeneous nature of disabilities within and across diagnostic categories likely contributes to variable responses to physical activity, with factors such as neurobiological differences, medication use, functional limitations, and sleep hygiene practices potentially moderating observed associations. Although multiple relevant databases were searched, the exclusion of EMBASE, CINAHL, and Web of Science may have resulted in omission of some eligible studies, and findings should be interpreted with this potential selection bias in mind. Additionally, because quantitative analyses were conducted using aggregated study-level data without variance-weighted modeling, the results are subject to potential bias and should not be interpreted as individual-level or causal effects, consistent with risks of ecological fallacy. Moreover, the quantitative synthesis is the inability to apply weighted regression techniques. Many included studies did not report variance estimates or effect sizes necessary for inverse variance weighting, and outcomes were operationalized inconsistently across studies. As a result, each study contributed equally to the regression models regardless of sample size, which may introduce bias and reduce precision. Although sample size was included as a covariate to assess its influence, the findings should be interpreted as exploratory, hypothesis-generating, and reflective of study-level trends rather than definitive estimates of association. Finally, it was limiting to dichotomize physical activity into meeting versus not meeting the 60 min daily guideline. This approach was necessary to harmonize highly heterogeneous physical activity measures across studies, which included objective accelerometry, self-report, proxy report, and guideline adherence metrics. However, dichotomization may result in information loss and measurement noise by collapsing diverse forms, intensities, frequencies, and contexts of physical activity into a single binary variable. Consequently, nuanced relationships between specific physical activity characteristics and sleep outcomes may not have been fully captured.

### 4.5. Lessons for Future Systematic Reviews

The present review also highlights challenges inherent in conducting systematic reviews in emerging, interdisciplinary fields. Of the 955 records identified, only 28 met inclusion criteria, reflecting substantial redundancy in the literature and the time-intensive nature of manual screening. While rigorous dual-reviewer screening remains the gold standard, future reviews may benefit from carefully implemented innovations such as AI-assisted title and abstract screening to improve efficiency without compromising methodological rigor. Emerging tools may help prioritize likely relevant records or reduce reviewer burden during early screening phases; however, transparency, validation, and human oversight remain essential. Thoughtful integration of such tools could support more sustainable review practices, particularly as public health literature continues to expand rapidly.

## 5. Conclusions

This review highlights the critical role of diagnosis type in influencing sleep duration among children and adolescents with disabilities, with individuals with physical disabilities typically experiencing longer sleep than peers with ASD, who exhibited the shortest durations. While the present review examined physical activity primarily in terms of guideline adherence based on duration, future research should investigate how additional dimensions of physical activity, including intensity, timing, and type, may relate to sleep outcomes across disability groups. However, given the diversity of disability-related functional and clinical profiles, physical activity recommendations should prioritize adaptability and safety rather than intensity alone. From a practice perspective, integrating structured, diagnosis-specific PA programs into treatment plans offers a promising strategy for improving sleep and overall health. Tailored approaches addressing barriers related to disability severity, age, and gender can enhance engagement, supporting holistic care and improved quality of life for this population.

## Figures and Tables

**Table 1:** Description of included studies (*N* = 28).

Author (Year)	*n*	Age (Years)	% Female	Diagnosis	Key Findings	PA–Sleep Association
Brand et al. (2015) [32]	10	10	50%	ASD	Aerobic exercise was associated with improved sleep efficiency, sleep onset, and wake time in children with ASD.	Positive
Cao et al. (2020) [33]	4178	14.3	53%	Depressive symptoms	Youth with low PA and poor sleep clustered with higher depressive symptom severity.	Not examined
Do et al. (2020) [34]	22	11	55%	Epilepsy	Exercise increased PA; marginal improvements in sleep quality were not related to PA changes.	Weak
Elkhatib Smidt et al. (2022) [35]	21,526	12.1	47%	ASD	PA was associated with greater odds of sufficient sleep duration; associations were weaker in autistic youth.	Weak
Garcia and Hahs-Vaughn (2021) [36]	894	12–17	NR	ASD	Youth with ASD more frequently exhibited low PA and poor sleep profiles than peers.	Not examined
Garcia et al. (2019) [37]	49	12.3	22%	ASD	Youth with better sleep quality engaged in more MVPA and less sedentary behavior.	Positive
Gerritsen et al. (2024) [38]	51	6.8	43%	Cerebral palsy	Higher MVPA was associated with shorter sleep duration the following night.	Negative
Haegele et al. (2020) [39]	561	13.8	56%	Visual impairment	Few youth met combined PA, sleep, and screen time guidelines.	Not examined
Healy et al. (2020) [14]	6821	10–17	NR	Multiple diagnoses	Youth with chronic conditions were more likely to not meet PA and sleep guidelines.	Not examined
Holton and Nigg (2020) [40]	184	10.3	29%	ADHD	Youth with ADHD were less likely to meet PA guidelines but more likely to meet sleep duration recommendations.	Not examined
Hong et al. (2021) [41]	309	9.4	26%	ADHD	PA partially mediated the association between ADHD diagnosis and sleep quality.	Positive
Hulst et al. (2023) [42]	54	3–12	44%	Cerebral palsy	Few children met both PA and sleep guidelines simultaneously.	Not examined
Kruizinga et al. (2020) [43]	12	2–31	25%	Intellectual disability	Youth with ID showed lower daily PA than peers; sleep and PA used as endpoints.	Not examined
Li et al. (2021) [44]	272	10–17	32%	ADHD	MVPA was positively correlated with sleep duration.	Positive
Loewen et al. (2020) [45]	3436	10–14	NR	ADHD	Most youth with ADHD did not meet PA or sleep duration recommendations.	Not examined
Maher et al. (2013) [46]	41	11–16	37%	Cerebral palsy	Youth with CP engaged in less PA than peers; no differences in sleep duration.	Not examined
McNarry et al. (2021) [47]	28	11.9	100%	Cystic fibrosis	Sedentary time was inversely associated with sleep duration; bidirectional effects observed.	Weak
Neville et al. (2021) [48]	118	2.7	28%	ASD	PA was associated with adaptive behaviors; sleep reported descriptively only.	Not examined
Pringsheim et al. (2021) [49]	110	10.9	22%	Tourette syndrome	Greater sedentary behavior and poorer sleep observed with increased symptom severity.	Positive
Ronen and Janssen (2019) [50]	163	14	51%	Epilepsy	Few youth met combined PA, sleep, and screen time guidelines.	Not examined
Siegel et al. (2020) [51]	54	8–14	NR	DMD	Sleep impairment associated with lower physical quality-of-life scores.	Not examined
Smit et al. (2020) [52]	36	15	39%	Cerebral palsy	Sedentary time was negatively correlated with sleep duration.	Weak
Tatsumi et al. (2015) [53]	31	4–6	19%	ASD	Higher PA earlier in the day was associated with improved sleep outcomes.	Positive
Tse et al. (2019) [29]	19	10.1	26%	ASD	PA intervention improved sleep efficiency, onset latency, and duration.	Positive
Vanhelst et al. (2013) [54]	410	15.1	46%	Intellectual disability	Longer sleep duration was associated with lower PA levels.	Negative
Wachob and Lorenzi (2015) [55]	10	11.8	NR	ASD	Higher MVPA was associated with less sleep disruption and earlier sleep onset.	Positive
Whitney et al. (2019a) [56]	111	6–17	37%	Cerebral palsy	Youth with CP were less likely to meet PA and sleep guidelines than peers.	Not examined
Whitney et al. (2019b) [57]	423	6–17	37%	Intellectual disability	Many youth with ID did not meet PA or sleep duration recommendations.	Not examined

Note: “Association” reflects whether a direct relationship between physical activity and sleep was statistically tested. “Not examined” indicates both variables were reported descriptively but not analyzed in relation to one another. In the studies included, PA was evaluated using both subjective and objective measures. Subjective assessments comprised questionnaires (*n* = 7), checklists (*n* = 2), and recall methods (*n* = 5). Objective measurements included study interventions (*n* = 3), where participants attended programs observed by researchers, and the use of actigraphy or other activity monitors (*n* = 13). Various PA outcomes were examined, such as whether participants met PA guidelines (*n* = 6), engaged in 60 min or more of PA in sports (*n* = 6), or were active for 60 min or more at school or afterschool (*n* = 4). Given the diversity in PA measurement and outcomes, the primary focus of this review was on whether participants met the guideline of 60 min or more of daily PA, which could be extracted or estimated from all the studies analyzed.

**Table 2:** Description of qualitative findings on PA and sleep by diagnosis (*N* = 14).

Diagnosis Category (*n* = studies)	Qualitative Finding	PA and Sleep Relationship
ASD (*n* = 4)	One study found that 70% of participants with ASD reported insomnia, but on nights after PA, sleep quality significantly improved (Brand et al., 2015 [32]). Another study had similar findings where, after a basketball intervention, participants with ASD reported improvements in sleep quality (Tse et al., 2019 [29]). High amounts of PA in the morning and afternoon can benefit sleep outcomes in youths with ASD (Tatsumi et al., 2015 [53]). However, the positive association between PA and sufficient sleep duration is weaker in autistic children, especially in those with more severe ASD, female autistic children, and autistic children aged 6–12 years old (Elkhatib Smidt et al., 2022 [35]).	Regular PA generally improves sleep quality and duration by regulating circadian rhythms and reducing stress. In children and youths with autism spectrum disorder (ASD), the benefits of PA on sleep are more variable and can be influenced by factors such as the severity of ASD, gender, and age.
Physical Disability (*n* = 5)	CP	CP
	Maher et al. (2013) [46] found children and youths with CP engaged in less physical activity, active transport, and team sports, with no significant differences in sleep compared to TD peers. Smit et al. (2020) [52] observed no significant correlation between active time and sleep quantity but found that more sedentary time was linked to less sleep. Hulst et al. (2023) [42] reported a negative bidirectional relationship between PA and sleep.	Participants with CP are generally less physically active than their typically developing peers but show no significant differences in sleep duration. There is also evidence of a negative bidirectional relationship between physical activity and sleep outcomes.
	CF	CF
	One study analyzed the effects of PA on sleep in adolescents with CF (McNarry et al., 2021) [47]. This study found a significant relationship between time spent being sedentary and poor sleep.	Increased PA may improve sleep quality in adolescents with CF.
	DMD	DMD
	DMD was explored in one study looking at the relationship between PA (using the 6-min walk test) and sleep (Siegel et al., 2020) [51]. This study found that rest-activity levels were directly correlated with subjective sleep impairment.	PA may have a small positive effect on sleep in children and youths with ambulatory DMD.
Neurological (*n* = 4)	ADHD	ADHD
	Recent studies have highlighted significant relationships between PA and sleep outcomes among individuals with ADHD. Li, Haegele, & Wang (2021) [44] identified a positive correlation between MVPA and improved sleep in adolescents with ADHD, contributing to enhanced school engagement. Additionally, Holton et al. (2020) [40] reported significant mediation effects where diet, PA, and screen time influenced ADHD’s impact on sleep quality, suggesting that interventions targeting these factors could enhance sleep quality in individuals with ADHD.	PA can potentially increase sleep outcomes in adolescents with ADHD. Other lifestyle factors should be considered when assessing the relationship between PA and sleep in adolescents with ADHD.
	Epilepsy	Epilepsy
	It was also found that those with epilepsy who did meet PA recommendations similar to their TD peers had better sleep outcomes (Do et al., 2020) [34]. However, changes in physical activity were not associated with changes in sleep outcomes when accounting for age, sex, and baseline	PA may improve sleep outcomes, but more research is needed.
	Tourette’s Syndrome	Tourette’s Syndrome
	Children with more severe tics had greater impairment in subjective sleep measures, more sedentary activity time, and less time engaged in light, moderate, and vigorous activities (Pringsheim et al., 2021) [49]. The results of this observational study indicate a small, but significant relationship between activity and sleep measures and the severity of the main symptom domains present in tic disorders	PA may improve sleep outcomes, but more research is needed.
ID (*n* = 1)	ID	ID
	One study found that those with ID who wake up earlier are more active than those who rise late. Participants with the greatest sleep duration were least active (Vanhelst et al., 2013) [54].	Sleep duration may play a role in the PA levels of individuals with ID. Interventions aiming to promote physical activity among individuals with ID should consider optimizing sleep patterns

Note: ASD = autism spectrum disorder, CP = cerebral palsy, CF = cystic fibrosis, DMD = Duchenne muscular dystrophy, ADHD = attention deficit hyperactivity disorder, ID = intellectual disability, TD = typically developing, PA = physical activity, MVPA = moderate-to-vigorous activity.

## Data Availability

The original contributions presented in this study are included in the article/Supplementary Materials. Further inquiries can be directed to the corresponding author.

## Supplementary Materials

The following supporting information can be downloaded at: https://www.mdpi.com/article/10.3390/ijerph23010121/s1. Classification of physical activity and sleep assessment methods (objective vs. subjective) for each included study.

## Abbreviations

The following abbreviations are used in this manuscript:

PA	Physical activity
MVPA	Moderate-to-vigorous physical activity
ASD	Autism spectrum disorder
ADHD	Attention-deficit/hyperactivity disorder
CP	Cerebral palsy
CF	Cystic fibrosis
DMD	Duchenne muscular dystrophy
DS	Down syndrome
ID	Intellectual disability
PRISMA	Preferred Reporting Items for Systematic Reviews and Meta-Analyses
