# Supplementary material for: Diagnosis-Specific Links Between Physical Activity and Sleep Duration in Youth with Disabilities: A Systematic Review with Quantitative Synthesis
Source: Int J Environ Res Public Health. 2026 Jan 19;23(1):121. doi: 10.3390/ijerph23010121 (PMC12840898; doi:10.3390/ijerph23010121)
Supplement: Supplementary file 1 [file ijerph-23-00121-s001.zip › ijerph-4089803-supplementary.pdf]

Manuscript Title: Diagnosis-Specific Links Between Physical Activity and Sleep in Youth with Disabilities: A Systematic Review and Meta-Analysis

Author: Janette M. Watkins, PhD, et al.

Journal: Disability and Health Journal

Instruction: Complete the right-hand column ('Location in Manuscript') to specify where each PRISMA item appears (page/section/figure).

Section/Topic	Checklist Item	Location in Manuscript
Title	Identify the report as a systematic review, meta-analysis, or both.	Title page, line 1
Abstract	Provide a structured summary including background, objectives, data sources, eligibility criteria, participants, interventions, study appraisal, synthesis methods, results, limitations, conclusions, and implications.	Abstract, lines 1–20
Introduction	Describe the rationale for the review in the context of what is already known.	Introduction, p. 2
Objectives	Provide an explicit statement of the objectives or questions being addressed.	Introduction, final paragraph, p. 4, lines 78-81
Methods	Specify inclusion and exclusion criteria for the review.	Methods – Eligibility Criteria, p. 4, lines 83-92
Methods	Describe information sources and search strategy.	Methods – Search Strategy, p. 5, lines 101-110
Methods	Specify the process for selecting studies (screening, eligibility).	Methods – Selection Process, p. 5, lines 111-117
Methods	Describe methods of data extraction and quality assessment.	Methods – Data Extraction & Quality Assessment, p 6, lines 112-128
Results	Provide results of study selection, ideally with a flow diagram.	Results – Figure 1

Results	Summarize study characteristics.	Results – Study Description, p. 6–7, Table 1
Results	Present results of individual studies and syntheses.	Results – Quantitative and Qualitative Analysis, p. 8–9, Table 2
Discussion	Summarize the main findings, including the strength of evidence and relevance to key groups.	Discussion, p. 10–12
Discussion	Discuss limitations of the evidence and of the review process.	Limitations, p. 13
Discussion	Provide a general interpretation of results in the context of other evidence, and implications for future research, policy, or practice.	Discussion, p. 12–14
Funding	Describe sources of funding and role of funders for the systematic review.	Title Page – Funding Statement
